# Supplementary material for: Human-correlated genetic models identify precision therapy for liver cancer
Source: Nature. 2025 Feb 19;639(8055):754–64. doi: 10.1038/s41586-025-08585-z (PMC11922762; doi:10.1038/s41586-025-08585-z)
Supplement: Supplementary file 2 — Reporting Summary [file 41586_2025_8585_MOESM2_ESM.pdf]

nature portfolio

Corresponding author(s): Miryam Müller, Thomas G. Bird

Last updated by author(s): 26/11/24

Reporting Summary

Nature Portfolio wishes to improve the reproducibility of the work that we publish. This form provides structure for consistency and transparency in reporting. For further information on Nature Portfolio policies, see our Editorial Policies and the Editorial Policy Checklist.

Statistics

For all statistical analyses, confirm that the following items are present in the figure legend, table legend, main text, or Methods section.

n/a	Confirmed	
☐	☒	The exact sample size (*n*) for each experimental group/condition, given as a discrete number and unit of measurement
☐	☒	A statement on whether measurements were taken from distinct samples or whether the same sample was measured repeatedly
☐	☒	The statistical test(s) used AND whether they are one- or two-sided<br>*Only common tests should be described solely by name; describe more complex techniques in the Methods section.*
☒	☐	A description of all covariates tested
☐	☒	A description of any assumptions or corrections, such as tests of normality and adjustment for multiple comparisons
☐	☒	A full description of the statistical parameters including central tendency (e.g. means) or other basic estimates (e.g. regression coefficient) AND variation (e.g. standard deviation) or associated estimates of uncertainty (e.g. confidence intervals)
☐	☒	For null hypothesis testing, the test statistic (e.g. *F*, *t*, *r*) with confidence intervals, effect sizes, degrees of freedom and *P* value noted<br>*Give P values as exact values whenever suitable.*
☒	☐	For Bayesian analysis, information on the choice of priors and Markov chain Monte Carlo settings
☒	☐	For hierarchical and complex designs, identification of the appropriate level for tests and full reporting of outcomes
☐	☒	Estimates of effect sizes (e.g. Cohen's *d*, Pearson's *r*), indicating how they were calculated

Our web collection on statistics for biologists contains articles on many of the points above.

Software and code

Policy information about availability of computer code

Data collection	No software was used
Data analysis	HALO image analysis (v3.1.1076.363), Columbus Image analysis (v2.8.0.138890), VivoQuant (v4.0), Icy BioImage, v2.0.0.0, GraphPad Prism software (v9), R (v 4.0.2 and higher), FastQC (v0.11.9), FastP (v0.20.1), MultiQC (v1.9), FastQ Screen (v0.14.0), STAR (v2.7.8a and v2.5.1b), Subread (v2.0.1), GenomicDataCommons (v1.12.0), GenePattern v3.9, maftools (v2.4.2), DESeq2, v1.28.1 and v1.44.0, biomaRt version 2.56.1, uwot(v0.1.11), RANN (v2.6.1), igraph, versions 1.2.11 and 2.0.3, corto, 1.2.4, msigdbr (v7.4.1), ComplexHeatmap v2.4.3, ggplot2 versions 3.3.6 and 3.5.1, cowplot (v1.1.1), clusterProfiler version 3.16.1, DIVA (v8.0.1), FlowJo (v9.9.6), Visiopharm (v2024.06.0.19093 x64 and v2024.07.1.16745 x64), Scribus (v1.4.8), Gimp (v2.10.14), Inform (v2.6.0), featureCounts (v1.5.2).<br><br>Code availability : Scripts used for disease positioning is available at https://codeocean.com/capsule/9804119/tree/v1

For manuscripts utilizing custom algorithms or software that are central to the research but not yet described in published literature, software must be made available to editors and reviewers. We strongly encourage code deposition in a community repository (e.g. GitHub). See the Nature Portfolio guidelines for submitting code & software for further information.

Data

Policy information about availability of data

All manuscripts must include a data availability statement. This statement should provide the following information, where applicable:

- Accession codes, unique identifiers, or web links for publicly available datasets
- A description of any restrictions on data availability
- For clinical datasets or third party data, please ensure that the statement adheres to our policy

Data files for transcriptomic analyses can be found on the Gene Expression Omnibus (GEO) repository; accession numbers: GSE275444 accessible via token 'ubefgeqkrxkjdep' on GSE273806 (Mouse models) and GSE275443 (Organoids). Our transcriptomic data are freely available to browse via a user-friendly interactive browsing online app enabling HuMo classification of external transcriptomic datasets (http://shinyapps.crukscotlandinstitute.ac.uk/humo_app/).
Immunohistochemical and H&E staining of the GEMMs are publicly available via BioImage Archive (https://www.ebi.ac.uk/) via accession number S-BIAD1365.
Montironi cohort data was provided upon request to the original authors (doi: 10.1136/gutjnl-2021-325918) and the TCGA data was accessed from publicly accessible databases (doi: 10.1016/j.cell.2017.05.046.). Mouse genome (GRCm39.103) was accessed from https://www.ensembl.org.
All data generated and/or analysed during the current study are also available from the corresponding authors on reasonable request.

Field-specific reporting

Please select the one below that is the best fit for your research. If you are not sure, read the appropriate sections before making your selection.

☒ Life sciences ☐ Behavioural & social sciences ☐ Ecological, evolutionary & environmental sciences

For a reference copy of the document with all sections, see nature.com/documents/nr-reporting-summary-flat.pdf

Life sciences study design

All studies must disclose on these points even when the disclosure is negative.

Sample size

No statistical methods were used to pre-determine sample sizes but our sample sizes are similar to those reported in previous publications. For animal experiments biological replicate sizes were chosen taking into account the variability observed in pilot and prior studies in respective cohorts. Animal studies were also carried out respecting the limited use of animals in line with the 3R system: Replacement, Reduction, Refinement

Data exclusions

No data were excluded without having met prespecified QC limits.
The following were excluded before analysis: One biological replicate failed QC post transcriptomic sequencing, all other biological replicates from this and other cohorts successfully passed QC and were included in downstream analysis; two drugs were excluded from the HCCO HTP screen due to microbiological contamination and drug precipitation in multiple replicates, respectively.
One sample was excluded from the RFP expression analysis during analysis (total n=4 biological replicates): testing AAV-mediated recombination of RFP alleles in females (ED Figure 1b), one sample was a notable outlier (4.9% vs 25.7/25.1/25.8%) which upon re-review was caused by inconsistent RFP staining of the section - this outlier was removed from final analysis; details are provided in the figure legend also.
Where tumour number could not be quantified due to tumour rupture no tumour number is reported (i.e. Fig 5d).

Replication

Individual animals of control and experimental cohorts are biologically unique - replicate data represents analysis of data/samples from independent replicate animals and is denoted by "n". Separate vehicle control treatment arms have been consistent with tumour penetrance and survival with the original untreated cohorts in all instances. Efficacy of cladribine and/or lenvatinib has been further replicated in a subsequent BM cohort. In vitro experiments were replicated at least once (HTP screen) but generally twice (dose validation assays) and results were replicable.

Randomization

To reduce the impact of confounding factors such as litter mates or induction dates for all experiments animals/sample assignment was matched for age-matched control and were assigned based upon randomly assigned mouse identification markings. No randomisation was performed during organoid screening or validation. Samples for transcriptomic analysis were prepared as a single batch sequentially without randomisation.

Blinding

The investigators were not blinded for the in vivo experiments. Technical staff administering therapy were blinded to the mouse genotypes. All subsequent tissue handling and analysis was blinded and/or performed using standardised automated analyses where possible.
Quantitative image analysis was performed blinded to the genotype and treatment.

Reporting for specific materials, systems and methods

We require information from authors about some types of materials, experimental systems and methods used in many studies. Here, indicate whether each material, system or method listed is relevant to your study. If you are not sure if a list item applies to your research, read the appropriate section before selecting a response.

Materials & experimental systems

n/a	Involved in the study
☐	☒ Antibodies
☐	☒ Eukaryotic cell lines
☒	☐ Palaeontology and archaeology
☐	☒ Animals and other organisms
☐	☒ Human research participants
☒	☐ Clinical data
☒	☐ Dual use research of concern

Methods

n/a	Involved in the study
☒	☐ ChIP-seq
☒	☐ Flow cytometry
☒	☐ MRI-based neuroimaging

Antibodies

Antibodies used

All antibodies used are also described in extended data table 5

Antibody/Manufacturer /Cat_no /Clone number (monoclonals)/ Lot number
GS Abcam ab49873 1/500 or 1/1000 NA GR3384613-5
GS Sigma-Aldrich HPA007316 1/1000 NA C81287
GS Sigma-Aldrich HPA007316 1/1000 NA C81287
Sox9 Milliore AB5535 1/500 NA 3249418
GFP Cell Signalling 2555 1/600 NA 6
HNF4a Santa Cruz SC6556 1/100 NA not available
HNF4a Santa Cruz SC374229 1/100 H-1 G0116
HNF4a Biotechne PP-H1415-00 1/500 H1415 A-2
RFP Tebu-Bio 600-401-379 1/1000 NA 42872
F4/80 AbD SeroTec MCA497 1/150 A3-1 GR3279416-2
CD68 Dako M0876 1/400 PG-M1 not available
CD4 eBioscience 14-9766-82 1/75 4SM95 2115647
CD4 Dako M7310 1/35 4B12 not available
CD8a eBioscience 14-0808-82 1/75 4SM15 2127137
CD8a AbD SeroTec MCA1817T 1/30 4B11 6088619
Ki67 Abcam ab16667 1/200 SP6 GR216200-3/ GR3313195-42
Ki67 Cell Signalling 12202 1/1000 D3B5 6
cleaved Caspase 3 Cell Signalling 9661 1/500 NA 45
CD3 Abcam ab16669 1/100 SP7 GR3247742-11
yH2AX Cell Signalling 9718 1/120 20E3 17
p53 Leica NCL-L-p53CM5p 1/150 NA 6087005
CD34 Biolegend 119302 1/100 Mec14.7 not available
Ctnnb1 BD BD610154 1 to 50 14 9315374
CD31 Abcam ab28364 1/75 NA 32447742-21
pAKT (473) Cell Signaling 4060 1/50 9DE 25
PTEN Cell Signaling 9559 1/70 138G6 19
Zeb1 Cell Signaling 70512 1/500 E2G6Y 2
c-Myc Roche Tissue Diagnostics 790-4628 RTU Y69 K05962
GS Sigma-Aldrich hpa007316 1/500 NA 16878
CD8 Thermo Fisher Scientific 14-0808-82 1 to 75 4SM15 2720194
CD4 Thermo Fisher Scientific 14-9766-82 1 to 25 4SM95 2526300
GranzymeB Novus Biologicals nb100-684 1/100 NA R705
GS Sigma-Aldrich hpa007316 1/500 NA 16878
CD45 Abcam ab10558 1/300 NA 1041690-2
Ctnnb1 Cell signalling Technologies 8480 1/25 D10A8 9
CD3e-BV650 (clone 17A2) BioLegend 100229 1/100 17A2 B394769
TCRβ-BV510 (clone H57-597) BioLegend 109234 1/100 H57-597 B367672
TCRd-FITC (clone GL3) eBioscience 11-5711-85 1/200 GL3 1935313
CD4-BV605 (clone GK1.5) BioLegend 100421 1/100 GK1.5 B386408
CD8a- BUV395 (clone 53-6.7) BD 563786 1/100 53-6.7 3003153
Granzyme B-AF647 (clone GB11) BioLegend 515406 1/50 GB11 B367007
PD1 BioLegend 114102 200ug RMP1-14 B411441
IgG isotype BioLegend 400502 200ug RTK2758 B409040

Validation

All antibody validation are also described in extended data table 5. All antibodies were used according to the manufacturer's intended application. Antibodies were also routinely validated within the lab and/or histology department on control tissue, with appropriate cellular/tissue localisation confirmed and where appropriate in cells morphologically consistent with the target and "No primary antibody (NPA)" controls were included for all stainings shown in this manuscript.

Antibody /det_species /Manufacturer /Cat_no /Application /Dilution /Notes /Clone number (monoclonals) /Lot number /Validation method/species/application
GS mouse Abcam ab49873 IF 1/500 or 1/1000 NA GR3384613-5 synthetic peptide by manufacturer, zonal specificity in liver by researchers/mouse/IHC
GS mouse Sigma-Aldrich HPA007316 IHC 1/1000 NA C81287 zonal specifity in liver by researchers/mouse/IHC

GS human Sigma-Aldrich HPA007316 IHC 1/1000 NA C81287 zonal specificity in liver by researchers/human/IHC
Sox9 mouse Milliore AB5535 IHC 1/500 NA 3249418 IHC and WB by manufacturer/mouse/IHC
GFP NA Cell Signalling 2555 IF 1/600 NA 6 transfected cells by manufacturer, specific expression in positive control tissue by researchers/NA/WB and IHC respectively
HNF4a mouse Santa Cruz SC6556 IF 1/100 discontinued - used Biotechne antibody subsequently NA not available hepatocyte specific nuclear expression by researchers/mouse/IHC
HNF4a mouse Santa Cruz SC374229 IF 1/100 H-1 G0116 hepatocyte specific nuclear expression by researchers/mouse/IHC
HNF4a mouse Biotechne PP-H1415-00 IF 1/500 H1415 A-2 isoform recognition by manufacturer, hepatocyte specific nuclear expression by researchers/human and mouse respectively/IHC
RFP NA Tebu-Bio 600-401-379 IF 1/1000 NA 42872 specific to purified RFP with no cross reactivity to human or mouse serum by manufacturer, specific expression in positive control tissue by researchers/NA/immunoelectrophoresis and IHC respectively
F4/80 mouse AbD SeroTec MCA497 IHC 1/150 A3-1 GR3279416-2 murine F4.80 antigen by manufacturer/mouse/IHC
CD68 human Dako M0876 IHC 1/400 PG-M1 not available clustered as anti-CD68 at Fifth International Workshop and Conference on Human Leucocyte Differentiation Antigens held in Boston in 1993/human/IHC
CD4 mouse eBioscience 14-9766-82 IHC 1/75 4SM95 2115647 cell treatment by manufacturer/mouse/IHC
CD4 human Dako M7310 IHC 1/35 4B12 not available none available
CD8a mouse eBioscience 14-0808-82 IHC 1/75 4SM15 2127137 relative expression by manufacturer/mouse/not available
CD8a human AbD SeroTec MCA1817T IHC 1/30 4B11 6088619 synthetic peptide by manufacturer/human/not available
Ki67 mouse Abcam ab16667 IF 1/200 SP6 GR216200-3/ GR3313195-42 knockout validated/mouse/IHC
Ki67 mouse Cell Signalling 12202 IHC 1/1000 D3B5 6 none available
cleaved Caspase 3 mouse Cell Signalling 9661 IHC 1/500 NA 45 blocking peptide by manufacturer/mouse/IHC
CD3 mouse Abcam ab16669 IHC 1/100 SP7 GR3247742-11 advanced validation by manufacturer/mouse/IHC
γH2AX mouse Cell Signalling 9718 IHC 1/120 20E3 17 DNA damage and lambda phosphatase +ve/-ve controls by manufacturer/ human/IHC
p53 mouse Leica NCL-L-p53CM5p IHC 1/150 NA 6087005 knockout by other researchers/mouse/WB
CD34 mouse Biolegend 119302 IHC 1/100 Mec14.7 not available none available
Ctnnb1 mouse BD BD610154 IF 1 to 50 14 9315374 murine immunogen and QC testing by manufacturer/human/WB
CD31 mouse Abcam ab28364 IHC 1/75 NA 32447742-21 endothelial localisation by manufacturer and researcher/human and mouse/IHC
pAKT (473) mouse Cell Signaling 4060 IHC 1/50 9DE 25 insulin treatment +ve and lambda phosphatase/human/IHC
PTEN mouse Cell Signaling 9559 IHC 1/70 138G6 19 knockout and control peptide by manufacturer/human/IHC
Zeb1 mouse Cell Signaling 70512 IHC 1/500 E2G6Y 2 recombinant protein/Human/NA
c-Myc mouse Roche Tissue Diagnostics 790-4628 IHC (duplex) RTU stain: anti-Rabbit HQ & anti-HQ HRP (Roche Tissue Diagnostics, 760-4815, 07017936001), Opal 570 (Akoya Biosciences, FP1488001KT), 1:50, 8 min Y69 K05962 CE-IVD antibody approved, a positive control (Human tonsil)was used to validate the assay and Ms liver staining was assessed by a pathologist; specificity of the secondary antibody was proved via negative control/Mouse and human/IHC
GS mouse Sigma-Aldrich hpa007316 IHC (duplex) 1/500 stain: OmniMap-antiRb HRP (Roche Tissue Diagnostics, 760-4311) 12 min, Opal 520 (Akoya Biosciences, FP1487001KT), 1:300, 8 min NA 16878 Antibody tested in WB and IHC by supplier, zonal specifity in liver by researchers, specificity of the secondary antibody was proved via negative control/mouse/IHC
CD8 mouse Thermo Fisher Scientific 14-0808-82 IHC (multiplex) 1 to 75 stain: OmniMap-antiRt HRP, 20 min (Roche Tissue Diagnostics, 760-4457), Opal 690 (Akoya Bioscеinces, FP1497001KT), 1:100, 8 min 4SM15 2720194 relative expression by manufacturer, immune cells in ms liver were assessed by a pathologist,specificity of the secondary antibody was proved via negative control /mouse/IHC
CD4 mouse Thermo Fisher Scientific 14-9766-82 IHC (multiplex) 1 to 25 stain: Impress anti-Rat, (Vector Laboratories, ZJ0512) 48 min, Opal 620 (Akoya Biosciences, FP1495001KT), 1:50, 8 min 4SM95 2526300 cell treatment by manufacturer, immune cells in ms liver were assessed by a pathologist,specificity of the secondary antibody was proved via negative control /mouse/IHC
GranzymeB mouse Novus Biologicals nb100-684 IHC (multiplex) 1/100 stain: OmniMap-antiRb HRP (Roche Tissue Diagnostics, 760-4311) 12 min, Opal 650 (Akoya Biosciences, FP1496001KT), 1:300, 8 min NA R705 Positive control (mouse spleen) was used to validate the assay and double positivity with CD8 was assessed by a pathologist in Ms liver,specificity of the secondary antibody was proved via negative control/mouse/IHC
GS mouse Sigma-Aldrich hpa007316 IHC (multiplex) 1/500 stain: OmniMap-antiRb HRP (Roche Tissue Diagnostics, 760-4311) 12 min, Opal 520 (Akoya Biosciences, FP1487001KT), 1:300, 8 min NA 16878 Antibody tested in WB and IHC by supplier, zonal specifity in liver by researchers, specificity of the secondary antibody was proved via negative control/mouse/IHC
CD45 mouse Abcam ab10558 IHC (multiplex) 1/300 stain: OmniMap-antiRb HRP (Roche Tissue Diagnostics, 760-4311), 12 min, Opal 480 (Akoya Biosciences, FP1500001KT), 1:25, 8 min NA 1041690-2 Antibody tested in WB and IHC by supplier, immune cells were assessed by a pathologist,specificity of the secondary antibody was proved via negative control /mouse/IHC
Ctnnb1 mouse Cell signalling Technologies 8480 IHC (multiplex) 1/25 stain: Ultramap-antiRb HRP (Roche Tissue Diagnostics, 760-4315) 12 min, TSA-DIG (Akoya Biosciences, FP1502001KT), 1:100, 12 min and Opal 780 (Akoya Biosciences, FP1501001KT), 1:10, 1h. D10A8 9 Antibody tested by supplier with HeLa Cells and tissue, zonal specifity in liver assessed by a pathologist, specificity of the secondary antibody was proved via negative control/human +mouse/WB and IHC
CD3e-BV650 (clone 17A2) mouse BioLegend 100229 FACS 1/100 17A2 B394769 manufacturer/mouse/FACS
TCRβ-BV510 (clone H57-597) mouse BioLegend 109234 FACS 1/100 H57-597 B367672 manufacturer/mouse/FACS
TCRd-FITC (clone GL3) mouse eBioscience 11-5711-85 FACS 1/200 GL3 1935313 none available
CD4-BV605 (clone GK1.5) mouse BioLegend 100421 FACS 1/100 GK1.5 B386408 manufacturer/mouse/FACS
CD8a- BUV395 (clone 53-6.7) mouse BD 563786 FACS 1/100 53-6.7 3003153 manufacturer/mouse/FACS
Granzyme B-AF647 (clone GB11) mouse BioLegend 515406 FACS 1/50 GB11 B367007 manufacturer/human+mouse/FACS
PD1 mouse BioLegend 114102 Therapy 200ug RMP1-14 B411441 The RMP1-14 antibody has been reported to block the binding of PD-1 to its ligands (B7-H1 and B7-DC) and to inhibit T cell proliferation and cytokine production costimulated by macrophages (but not by dendritic cells and B cells)
IgG isotype mouse BioLegend 400502 Therapy control 200ug RTK2758 B409040 screening of a variety of resting, activated, live and fixed mouse tissues by manufacturer/mouse/IHC

Eukaryotic cell lines

Policy information about cell lines

Cell line source(s)	Human organoid cell lines were previously described (Nuciforo et al., Cell Reports, 2018). Murine organoid lines were derived at the Cancer Research UK Beatson Institute.<br>The Hep-53.4 (RRID:CVCL_5765) was purchased from Cytion – LOT-L230232R
Authentication	None of the murine cell lines were authenticated as they were mouse-derived organoid lines. Comparison, both histological, protein expression and bulk transcriptome is provided in comparison to originating tumour models in the manuscript.<br>The same Hep53.4 line was used in house for this study as was used previously (doi: 10.1136/gutjnl-2021-326259) including genomic and transcriptomic characterisation at that time.
Mycoplasma contamination	lines were routinely tested by PCR for mycoplasma contamination and the lines used tested negative.
Commonly misidentified lines (See ICLAC register)	no commonly misidentified lines were used in this study

Animals and other organisms

Policy information about studies involving animals; ARRIVE guidelines recommended for reporting animal research

Laboratory animals	Details for all animals involved in this study can be found in the methods section and are as follows:<br>Unless otherwise specified male mice on a mixed background were used. The following transgenic mice strains were used: Gt(ROSA)26Sortm14(CAG-tdTomato)Hze (R26LSL-Tom), Ctnnb1tm1Mmt (Ctnnb1ex3), Gt(ROSA)26Sortm1(MYC)Djmy (R26LSL-MYC), Trp53tm1Brn (Trp53fl), Trp53tm2Tyj (Trp53R172H), Cdkn2atm1.1Brn (Cdkn2aKO), Ptentm2Mak (Ptenfl), Gt(ROSA)26Sortm1(Notch1)Dam (R26LSL-NICD), Krastm4Tyj (KrasG12D), Cdkn1atm1Led (Cdkn1aKO), Axin1 (Axin1fl) and Bap1tm2c(EUCOMM)Hmgu. Mice were induced between 8 and 12 weeks of age, unless otherwise indicated.<br>For the GEMM+MWD model 6-week old mice were kept on a modified western diet (Envigo -TD.120528) plus sugar water (23.1 g/L fructose and 18.9 g/L glucose) in combination with repeated CCl4 injections (ip, 0.2 µl/g of body weight, Veh: Cornoil) as referenced and were induced with AAV-TBG-Cre at 10 weeks of age.<br>For the DEN/ALIOS model, C57BL/6 WT mice, were injected with a single dose of DEN (80 mg/kg by i.p. injection) at 14 days of age. Mice were fed ALIOS diet (Envigo, TD.110201) and sugar water (23.1 g/L fructose and 18.9 g/L glucose) from 60 days of age. Mice were harvested at day 284.<br>For MWD+CCl4 model mice were kept on a modified western diet (Envigo -TD.120528) plus sugar water (23.1 g/L fructose and 18.9 g/L glucose) in combination with repeated CCl4 injections (ip, 0.2 µl/g of body weight, Veh: Cornoil) as previously described51.<br>For the streptozotocin (STZ) model, male and female C57BL/6J WT mice were injected with a single dose of STZ (200µg in 0.1M citrate buffer, pH 4.0) subcutaneously at 2 days of age. Mice were fed high-fat diet (TestDiet 58R3, cat.no. 1810835) from 30 days of age.<br>For the orthotopic model, Hep-53.4 cells (female C57BL/6J hepatoma cell line) were injected intrahepatic into the left lobe of male C57BL/6J mice.<br>Mice were housed under controlled conditions (specific pathogen free, 12hr light-dark cycle, 19-22 °C, 45-65% humidity) with access to food and water ad libitum. We added environmental enrichments, in the form of gnawing sticks, plastic tunnels, and nesting material to all cages.
Wild animals	no wild animals were used in this study
Field-collected samples	no field-collected samples were used in this study
Ethics oversight	All animal experiments were performed in accordance with UK Home Office licences (70/8891, PP0604995, 70/8646, 70/8468, and PP8854860) and in accordance with the UK Animal (Scientific Procedures) Act 1986 and EU direction 2010. They were subject to review by the animal welfare and ethical review board of the University of Glasgow and the University of Newcastle upon Tyne

Note that full information on the approval of the study protocol must also be provided in the manuscript.

Human research participants

Policy information about studies involving human research participants

Population characteristics	All human TCGA data used in this study is publically available (doi: 10.1016/j.cell.2017.05.046). HuMo clusters were validated with the bulk RNAseq data of an independent cohort of 171 HCC samples from patients undergoing resection collected in the setting of the HCC Genomic Consortium (European Genome-Phenome Archive code EGAS00001005364).
Recruitment	No patients were recruited for this study.
Ethics oversight	For the representative human HCC sample (no longer included post revision): the use of consenting patients' tissues surplus to diagnostic requirements for research purposes was approved by the Newcastle and North Tyneside Regional ethics committee, the Newcastle Academic Health Partners Bioresource (NAHPB) and the Newcastle upon Tyne NHS Foundation Trust Research and Development (R&D) department, in accordance with Health Research Authority guidelines. (References 10/H0906/41; NAHPB Project 48; REC 12/NE/0395; R&D 6579; Human Tissue Act license 12534).<br>Ethics oversight for the previous cohorts described in the TCGA and Montironi et al. cohorts have been described previously.

Note that full information on the approval of the study protocol must also be provided in the manuscript.
